# Role of pH-sensing receptors in colitis

**DOI:** 10.1007/s00424-024-02943-y

**Published:** 2024-03-22

**Authors:** Martin Hausmann, Klaus Seuwen, Cheryl de Vallière, Moana Busch, Pedro A. Ruiz, Gerhard Rogler

**Affiliations:** Department of Gastroenterology and Hepatology, University Hospital Zurich, University of Zurich, 8091 Zurich, CH Switzerland

**Keywords:** Inflammatory bowel disease, pH-sensing G protein-coupled receptors, GPR65, TDAG8, GPR4, GPR68, OGR1, Fibrosis, Colitis-associated cancer, EndoMT, Hypoxia

## Abstract

Low pH in the gut is associated with severe inflammation, fibrosis, and colorectal cancer (CRC) and is a hallmark of active inflammatory bowel disease (IBD). Subsequently, pH-sensing mechanisms are of interest for the understanding of IBD pathophysiology. Tissue hypoxia and acidosis—two contributing factors to disease pathophysiology—are linked to IBD, and understanding their interplay is highly relevant for the development of new therapeutic options. One member of the proton-sensing G protein-coupled receptor (GPCR) family, GPR65 (T-cell death-associated gene 8, TDAG8), was identified as a susceptibility gene for IBD in a large genome-wide association study. In response to acidic extracellular pH, GPR65 induces an anti-inflammatory response, whereas the two other proton-sensing receptors, GPR4 and GPR68 (ovarian cancer G protein-coupled receptor 1, OGR1), mediate pro-inflammatory responses. Here, we review the current knowledge on the role of these proton-sensing receptors in IBD and IBD-associated fibrosis and cancer, as well as colitis-associated cancer (CAC). We also describe emerging small molecule modulators of these receptors as therapeutic opportunities for the treatment of IBD.

## Inflammatory bowel disease (IBD) is accompanied by low pH in the gut

IBD includes two main phenotypic subgroups: ulcerative colitis (UC) and Crohn’s disease (CD). In 2017, 6.8 million cases of IBD were documented worldwide [16]. Chronic intestinal wall inflammation that results in severe and long-lasting mucosal tissue destruction is a defining feature of IBD. An expanding body of literature has provided evidence that the pathophysiology of IBD is associated with genetic susceptibility [34], intestinal microbiota alterations [73], environmental factors [114], and immunological abnormalities [90].

Increased local proton concentration, tissue hypoxia, low perfusion, and high levels of glycolytic metabolite synthesis (e.g., lactic acid) reduce tissue pH in chronic inflammation and inflammation-associated malignancies [5, 29, 69, 94, 105], leading to pro-inflammatory cytokine production [69]. An acidic tissue microenvironment is not only the result of inflammation but also influences the degree and outcome of inflammation [8, 44, 88]. In line with this, the pathogenesis of IBD as well as disease flares have been linked to a local decrease of the pH in the gut lumen and surrounding mucosa, with some reports showing a pH ranging between 2.3 and 3.4 in the proximal colon of patients with active UC [29, 94]. Caprilli et al*.* also showed that the fecal fluid is characterized by low pH and bicarbonate in patients with severe UC when compared to healthy controls (pH, 6.06 versus 6.52; HCO3^−^, 8.4 versus 34.6 mq/L) [11]. Moreover, an early study which monitored changes in lactate levels in relation to UC severity reported a progressive increase in lactate, from mild colitis (3.0 ± 1.8 mM/L) to severe colitis (21.4 ± 18.6 mM/L) [139].

About 20 years ago, a family of G protein-coupled receptors (GPCRs) was described to sense extracellular protons in a narrow and physiologically relevant pH range. The G protein-coupled receptor (GPR)4 family of proton-sensing receptors consists of three members: GPR4, GPR65 (T-cell death-associated gene 8, (TDAG8) and GPR68 (ovarian cancer G protein-coupled receptor 1, OGR1). They are activated by a decrease in extracellular pH (below the physiological level), reaching their maximal activation at around pH 6.8. In contrast, they are inactive or almost silent at a pH higher than 7.6 [78, 88]. These receptors were shown to play important roles in inflammation, bone metabolism, pain-sensing, kidney function, and breathing regulation [51, 54, 123].

With respect to IBD, three aspects make research on pH-sensing receptors particularly interesting. First: pH-sensing receptor TDAG8 has been identified as an IBD risk gene in genome-wide association studies [34]. Second: Specific pH-sensing receptors that are known to play a role in inflammatory processes can be detected in the intestine. Third: Orally active antagonists that selectively bind to pro-inflammatory pH-sensing receptors (OGR1, GPR4) have been recently developed.

It should be noted that a fourth receptor, G2A/GPR132, was described as pH sensing [89]. However, effects were cell-type-dependent, and similar evidence could not be obtained by other laboratories [106, 123]. This receptor also lacks key amino acid residues required for pH sensing in GPR4, OGR1, and TDAG8 [116]. There is strong evidence today that G2A/GPR132 is activated by oxidized lipids and other related molecules [33]. For these reasons, we do not consider G2A/GPR132 as a bona fide pH-sensing receptor, and we limit the scope of this review to GPR4, OGR1, and TDAG8. We provide here an overview of the association of these GPCRs with inflammation, fibrosis, and tumorigenesis in the context of IBD.

## Proton-sensing G protein-coupled receptors are linked to IBD

### TDAG8

TDAG8 is mainly expressed in cells of the immune system [134]. In humans, TDAG8 is expressed predominantly in peripheral blood leukocytes and lymphoid tissue, including the spleen, lymph nodes, and thymus [67], suggesting an important function in innate and adaptive immune reactions. TDAG8 acts as a negative regulator of inflammation [95, 131] through the activation of a Gα_s_-coupled mechanism [88], which augments downstream cyclic adenosine monophosphate (cAMP). Upon extracellular acidification, TDAG8 activates the adenylyl cyclase (AC)/cAMP/Protein Kinase A pathway through Gs proteins [54, 140] and Rho signaling via G_12/13_ [54]. Rho signaling is of major importance in controlling leukocyte migration and phagocytosis in innate immune cells.

Interest in the role of TDAG8 in the gut increased when a large-scale meta-analysis identified a single nucleotide variant in the *TDAG8* gene (I231L, rs3742704) as a susceptibility locus for IBD [56]. Additionally, the minor *TDAG8* variant rs8005161 is associated with UC [130], and IBD patients carrying the rs8005161-TT and rs8005161-CT alleles present an increased disease severity [130]. Following an extracellular acidic pH shift imposed on CD14^+^ monocytes from IBD patients, an impaired RhoA activation was observed irrespective of the rs8005161 allele [130], suggesting a still unknown adaptation of this signaling pathway in the disease.

In mice, TDAG8 is found on mast cells in the jejunum [154], T cells, macrophages, dendritic cells, and granulocytes in the *lamina propria* of the colon [131]. In inflamed tissue, the expression of TDAG8 is increased compared to non-inflamed areas, likely reflecting increased leukocyte influx [131]. The function of TDAG8 has been investigated in *Tdag8* knock-out mice as well as knock-in mice harboring the murine orthologue of the IBD-associated variant GPR65 I231L. These strategies result in loss of function or diminished function of TDAG8, respectively. Loss of functional TDAG8 in *Tdag8* knock-out mice increases the recruitment of macrophages and neutrophils to the colon and enhances the expression of pro-inflammatory mediators in murine models of acute and chronic colitis, such as the DSS model of colitis, the T cell transfer model [81, 131], or interleukin (IL)-10-deficient animals [102]. In the latter model, the pro-inflammatory effect of TDAG8 deficiency is particularly pronounced and manifests with a significant increase in IL-6 levels and an increased presence of F4/80^+^CD64^+^ macrophages and IL-23^+^γδT cells in colitis lesions [102]. IL-10 is an important immune regulator in the gut, promoting mucosal tolerance and preserving epithelial integrity [91]. The available data indicate that TDAG8 signaling cooperates with IL-10 signaling to maintain homeostasis. A role of TDAG8 beyond the regulation of inflammatory mediators and lymphocyte trafficking is found in pathogen clearance through lysosomal activity. Deletion of *Tdag8* in mice or knock-in of the IBD-associated variant GPR65 I231L results in lysosomal dysfunction, which impacts bacterial autophagy and pathogen defense, thereby increasing the risk of developing colitis [70]. Thus, functional TDAG8 was shown to play an important role in the clearance of *Citrobacter rodentium* in a murine infection model [70].

Th17 cells and their secreted cytokines play an important role in the abnormal immune response in IBD [14]. Activation of TDAG8 is linked to Th17- and Th22-cell differentiation, and RAG1^−/−^ mice reconstituted with TDAG8-deficient T cells were protected from experimental autoimmune encephalitis (EAE) [37]. Impaired TH17 cell differentiation in TDAG8-deficient mice was also reported in the T cell transfer model of colitis and correlated with elevated IL12 and IL23 [13]. IL23 and its receptor IL23R play a key role in IBD pathogenesis [84], and IL17 may have a protective role in IBD rather than a detrimental one as in psoriasis or EAE [30]. In addition to IBD, TDAG8 has been identified as a risk gene for other inflammatory diseases, such as chronic obstructive pulmonary disease, asthma, multiple sclerosis, and ankylosing spondylitis [45, 47, 53, 56].

### GPR4

Initial reports described an important role of GPR4 in endothelial cell (EC) function, and sphingosylphosphorylcholine [62] and lysophosphatidylcholine [79] were described as ligands of GPR4. Although these molecules are no longer considered specific ligands of GPR4, there is a high correlation between the transcriptional regulation of GPR4 and endothelial function [26, 86, 148]. Open public repositories of gene expression data report moderate expression of GPR4 in the small intestine and colon. IBD patients exhibit increased GPR4 mRNA expression compared to healthy controls [141], which most likely reflects inflammation accompanied by an angiogenic response. Additionally, single-cell RNA sequencing (scRNA-seq) revealed that, in the human colon, GPR4 is expressed in CD36^+^ ECs present in the capillaries and in Duffy antigen/receptor for chemokines (DARC)^+^ cells lining postcapillary venules [65].

In mice, *Gpr4* mRNA has been detected on ECs in the *lamina propria* of the colon [143]. This was confirmed in scRNA-seq profiles where GPR4 was found in the colon arteries, arterioles, capillaries, venules, and vein cells [55]. Deletion of GPR4 in mouse models of experimental colitis decreased the expression of the endothelial adhesion molecules, vascular cell adhesion molecule-1 (VCAM1), and E-selectin in the intestinal microvasculature. This was associated with decreased mucosal leukocyte infiltration and reduced intestinal inflammation [117, 141]. Intestinal inflammation in CD is indeed characterized by an increased vessel density and angiogenesis [20, 119], associated with enhanced production of vascular endothelial growth factor (VEGF) in the local microvasculature. These data are in agreement with the notion that GPR4 drives VEGF production and is required for a full angiogenic response to this growth factor [148].

Similar to TDAG8, GPR4 is a Gs-coupled receptor which drives the activity of adenylate cyclase to generate the second messenger cAMP [78]*.* Signaling via G_13_ and G_q/11_ has also been documented, resulting in the activation of RhoA signaling pathway and calcineurin-dependent nuclear factor of activated T cell (NFAT)1 promoter-driven transcription, respectively [133]. In ECs, Rho family proteins are of particular importance in regulating endothelial barrier function and leukocyte transmigration [125].

While expression of GPR4 appears prominent in ECs, there is also evidence for its presence in other cell types. Colon tissue scRNA-seq profiling from both murine [55] and human [65] cellular communities revealed the presence of GPR4 also in stromal cells, including pericytes surrounding vessels and fibroblasts. In our own work, we detected GPR4 mRNA expression in murine and human primary intestinal fibroblasts [143]. The increased number of GPR4^+^ cells in intestinal inflammation may not only arise through local angiogenesis and recruitment of precursor cells but also through mechanisms involving transition from ECs or pericytes and cell de-differentiation [107]. For instance, it is well documented that inflammation can result in endothelial-to-mesenchymal transition (EndoMT) [109] and could contribute to an increased number of GPR4^+^ mesenchymal cells. EndoMT may be driven by the pro-inflammatory cytokines tumor necrosis factor (TNF)α and IL-1β [12], as well as pro-fibrotic transforming growth factor (TGF)-β1 [3, 152], a powerful immune mediator upregulated in IBD.

Pericytes surrounding the vascular system also represent a cellular reservoir for several mesenchymal cell types during tissue repair [38]. These cells can detach from vessels and differentiate into collagen-synthesizing fibroblasts, as observed in experimental dermal scarring [126]. Importantly, VEGF signaling stimulates myofibroblast transformation through the induction of TGFβ1, as shown for subconjunctival [100] and kidney fibrosis [76]. These data indicate that GPR4 could represent a target for the treatment of fibrotic diseases. In this context, it is important to note that pharmacological tools already exist to inhibit GPR4, and these molecules have been successfully tested in several animal models, including models of colitis ([86, 118, 143], and chapter below.)

### OGR1

In contrast to the rather cell type-specific expression of TDAG8 and GPR4, predominant in immune system cells and in ECs, respectively, OGR1 is expressed in various cell types, including monocytes/macrophages, T cells, granulocytes, ECs, and various mesenchymal cell lineages. OGR1 couples predominantly through Gα_q/11_ proteins, leading to activation of the phospholipase C (PLC)/inositol phosphate (IP)/Ca^2+^/extracellular signal-regulated kinases (ERK) pathway [78] and the Gα_12/13_/Rho signaling pathway [77, 134, 144, 146]. A role of OGR1 in inflammation was described early on, and several studies have shown a crucial role for OGR1 in the expression of inflammatory and tissue remodeling factors under acidic conditions [49, 83]. In IBD patients, intestinal tissue shows increased expression of OGR1, especially in inflamed areas, compared to the intestinal mucosa of healthy individuals [22, 25]. Furthermore, the expression of OGR1 correlates with higher clinical scores in IBD patients, suggesting that OGR1 has a clinically relevant pro-inflammatory effect that could be targeted for treatment [22, 25]. In a murine model of spontaneous colitis, OGR1 perpetuated intestinal inflammation through the expression of IL-6, TNFα, IL-8, and SPARC [22], a collagen-binding protein that mediates fibrosis [6, 7], while *Ogr1*-deficiency was protective [22, 25].

The extracellular acidification-induced expression of these OGR1-dependent genes was greatly enhanced under hypoxic conditions in human intestinal macrophages, and this effect was reversed by NF-κB inhibition [23]. OGR1 has also been implicated in other physiological processes, including kidney function and bone metabolism [50, 66]. Of particular interest is the recent observation that a homozygous loss of function of OGR1 was described in families with amelogenesis imperfecta, suggesting that OGR1 is required for dental enamel formation [101]. At present, there is no information on the response of these individuals to inflammatory challenges, but this observation suggests that therapeutic inhibition of OGR1 may be well tolerated in adult subjects.

## Hypoxia regulates the expression and function of pH-sensing receptors

Accumulating evidence shows that the intestinal mucosa and the underlying tissue are deprived of adequate oxygen supply during inflammation [2, 27, 59]. Hypoxia results from increased local metabolic requirements from resident and infiltrating inflammatory cells combined with a reduced oxygen supply from the bloodstream due to edema, vasoconstriction, and thrombosis [15, 59, 64].

Adaptive transcriptional responses to oxygen tension are mediated through the hydroxylation of the nuclear factors hypoxia-inducible factor (HIF)-1α and HIF-2α. Oxygen deprivation blocks hydroxylation, allowing HIF-1α and HIF-2α to accumulate and translocate to the nucleus where the expression of HIF target genes is induced [122]. In accordance with the hypoxic conditions in inflamed tissue, both HIF-1α and HIF-2α are induced in the mucosa from IBD patients and mouse models of colitis [39, 59]. HIFs can also be stabilized following the activation of immune receptors, such as Toll-like receptors (TLRs) [103], or by metabolic by-products, such as succinate [127], which signals through GPR91. HIFs are also stabilized during infection with different pathogens under hypoxic conditions [60, 93, 103, 145] and through hypoxia-independent mechanisms [42], including the inhibition of prolyl hydroxylase domain proteins through the chelation of Fe^2+^ ions with bacterial siderophores [46]. Thus, tissue hypoxia plays an important role in the regulation of innate immunity and inflammatory responses. At a mechanistic level, hypoxia activates the NF-κB signaling pathway [27, 128] by blocking the hydroxylation of upstream IκB kinase (IKK)-β [19]. Additionally, hypoxia triggers the expression of pro-inflammatory TNFα and IL-6 in monocytes [23] and supports inflammasome activation and production of IL-1β [32].

In accordance with the hypoxic environment and increased acidosis observed in IBD [39, 121], low oxygen tension promotes the expression of pH-sensor OGR1 via HIF-1α in human monocytic cell lines, intestinal epithelial cells, and macrophages, suggesting a crucial role of OGR1 in hypoxia-associated responses [23]. Additionally, TNFα induces the expression of OGR1 in cells of human macrophage lineage and primary human monocytes through an NF-κB-mediated mechanism [22, 23]. A study using rabbits subjected to hypoxia showed that TNFα is able to induce the binding of HIF-1α to the promoter of HIF-1α target genes through a NF-κB-mediated mechanism [135]. An in silico analysis identified several putative DNA binding sites for NF-κB and HIF-1α within the proximal regions of the OGR1 promoter variants [22]. Accordingly, chromatin precipitation analysis showed that hypoxia induced the binding of HIF-1α to the OGR1 promoter, confirming that OGR1 is under the transcriptional control of HIF-1α.

Recent reports indicate that low oxygen partial pressure experienced during aircraft travel heightens the risk of flares in IBD patients [138], and altitude-associated hypoxia increases pro-inflammatory gene expression in the duodenum of healthy subjects [147]. Accordingly, the expression of OGR1 is upregulated in the intestinal mucosa of CD and UC patients subjected to hypoxic conditions resembling an altitude of 4000 m above sea level for 3 h [23]. Considering that the intestinal epithelium is constantly exposed to luminal insults and oxygen deprivation, the interdependence between HIF and NF-κB signaling pathways appears most important in regulating intestinal epithelial barrier endurance and function [35, 129], and OGR1 may play a crucial effector role in inflammation driven by hypoxia and acidosis.

## Intestinal fibrosis as a consequence of inflammation/acidification

An exquisite balance between multiple pro- and anti-fibrotic stimuli on extracellular matrix (ECM)-producing cells is necessary for adequate wound healing [61, 98, 136, 137]. Rapid wound closure is crucial to reduce the time of an impaired barrier function of the intestinal wall. Nevertheless, the recurring and sometimes excessive tissue repair caused by inflammation leads to fibrosis, which may impair gastrointestinal function. Intestinal fibrosis is a common clinical problem in patients with CD and UC [21] that leads to stricture formation requiring surgical intervention in 30–50% of CD patients [18, 71, 108]. In the context of CD, clinical findings have shown that fibrosis only develops in segments of the gut where inflammation is present [110].

Fibroblasts are central to tissue fibrosis because they are key producers of deposited ECM components and possess contractile abilities and the capacity to secrete growth factors [36]. Fibroblasts in fibrotic tissue can arise in multiple ways: Fibroblasts may migrate into the inflamed area, local stromal fibroblasts may proliferate, and importantly, ECs and epithelial cells may undergo de- and re-differentiation processes, namely EndoMT and epithelial to mesenchymal transition (EMT), respectively [9, 107]. EMT is a well-characterized phenomenon that takes place in different tissues and diseases [92]. TGF-β1 is a key inducer and an important regulator of EMT. Upon exposure to inflammatory stimuli (e.g., cytokines [74, 75], extracellular matrix components [10, 75], pathogen-associated molecular patterns [72], and damage-associated molecular patterns [31] such as succinate [127, 149]), fibroblasts switch to an activated state. Of particular interest here, acidification can be added to the list of inflammatory stimuli.

OGR1 expression and OGR1-dependent signaling were observed in primary human and murine intestinal fibroblasts, and proton-activated OGR1-mediated signaling increases filamentous actin (F-actin) stress fibers at acidic pH in vitro [25]. *Gpr4* expression has also been reported in primary intestinal fibroblasts [143], and fibroblasts found in other organs, e.g., rat kidney [111]. Baseline levels of GPR4 may already be sufficient to promote inflammation or fibrosis. Interestingly, an increased expression of GPR4 was determined in primary intestinal fibroblasts following an acidic pH shift in vitro [143]. The fact that *GPR4* mRNA expression is detected in human and murine primary intestinal fibroblasts and that the cells respond to acidification with increased activation of RhoA, an important mediator of fibrosis [143] suggests that GPR4^+^ ECs are converted to GPR4^+^ fibroblasts by EndoMT.

During the course of kidney fibrosis in mice, approximately 14–15% of fibroblasts are derived from the bone marrow and about 36% emerge via EMT [58]. Approximately 30 to 50% of fibroblasts showed co-expression of EC and fibroblast markers [153] and may be derived from ECs residing within the kidney via EndoMT. The remaining fibroblast populations may arise from resident fibroblasts, pericytes [97, 104], and fibrocytes in the circulation [1]. It is plausible that fibroblast recruitment in intestinal inflammation follows a similar pattern.

In human disease, paired intestinal tissue samples from patients with CD undergoing ileocecal resection due to stenosis were analyzed. Samples from highly fibrotic areas showed an increased expression of *GPR4* as compared to the non-fibrotic areas [143]. *GPR4* and the expression of markers involved in different phases of fibrosis, e.g., ACTA2, a marker for myofibroblast activation, and the pro-collagens *COL1A1* and *COL3A1*, were positively correlated [143]. Similarly, samples from fibrotic areas presented higher expression levels of *OGR1* as compared to the non-fibrotic resection margin, and the expression of fibrotic markers, such as ACTA2 and the pro-collagens *COL3A1* and *TGFβ1* were positively correlated [48]. One can speculate that elevated numbers of GPR4^+^ and OGR1^+^ fibroblasts in intestinal fibrosis may arise through EndoMT, with GPR4 being inherited from the parental ECs. Increased expression of GPR4 and OGR1 triggered by inflammation-associated acidification, and subsequent cellular responses, may perpetuate inflammation-induced fibrosis in IBD. Similar considerations apply for the development of fibrosis in severe asthma and irreversible airway obstruction [63, 83].

In mice, *Gpr4*-deficiency leads to a decrease in fibrogenesis in models of intestinal fibrosis [143]. The presence of shorter vessels and decreased angiogenic factors were indicative of lowered vascularization. Additionally, mRNA expression levels of *Pdgfβ*, a potent chemoattractant for fibroblasts and other cells [115], were reduced in *Gpr4*^*−/−*^ mice. Likewise, decreased fibrosis was observed in *Ogr1*-deficient mice following chronic or spontaneous colitis. *Ogr1*-deficiency leads to a decrease in mRNA expression of fibrosis markers, as well as a reduction of collagen deposition in models for intestinal fibrosis [48]. In summary, the absence of GPR4 or OGR1 is associated with a decrease in inflammation [22, 141] and fibrosis [48, 143] in animal models of intestinal fibrosis.

## Proton-sensing GPCRs in colorectal cancer (CRC)

CRC is the second most diagnosed cancer in women and the third in men. Different risk factors contribute to the development of CRC, such as the patient’s genetic background, age, diet, environmental factors, as well as chronic intestinal inflammation as in CD or UC, and colitis-associated cancer (CAC). The risk of CAC in patients with IBD is increased after a long disease duration, especially in patients with chronic active disease [41, 113]. Oncogenic mutations alone do not seem sufficient to induce CRC or CAC. Tumorigenesis, tumor growth, and the formation of metastases depend on additional, non-mutational mechanisms. Such mechanisms involve inflammatory or regenerative programs that are either activated in the tumor tissue itself or in cells of the surrounding tissue stroma [40]. These programs induce phenotypic plasticity in CRC cells such as EMT [40], a prerequisite for fistula formation [4] and fibrosis in IBD [120], as well as metastasis [87].

Hypoxia, inflammation, fibrosis, and tumor formation in the intestine, as in CRC and CAC, are linked on many levels and induce each other [27]. Thus, hypoxia may be induced by inflammation (inflammatory hypoxia) [27], and it influences tissue pH in the mucosa [96]. It is well established that an acidic environment is not only an epiphenomenon that results from tumor formation or inflammation but also affects the outcome of the immune response to cancers [8, 44, 88]. Acidosis modulates EMT [132] and changes the expression of EMT cell markers in vitro and in vivo [112].

In tumors, protons (H^+^) are generated and accumulate due to several mechanisms, including increased anaerobic and aerobic glycolysis (Warburg effect). As a result, lactic acid is released and acidification of the tumor-associated extracellular micro-environment (TME) follows [69, 99, 124].

In the TME, a local pH below 7.0 is not uncommon and contributes to the progression of malignancy, tumor growth, metastasis, metabolic rewiring, and a decreased immune surveillance [17, 28, 57, 68]. Consequently, restoring normal tissue pH has been suggested to reduce tumor growth and to improve anti-tumor therapy [142]. However, normalizing the pH is difficult to achieve in the cancer tissue microenvironment. It is therefore tempting to consider the pH-sensing GPCRs, which mediate at least some effects of low pH on cells and tissues, as targets for therapy.

Proton-sensing GPCRs, TDAG8, GPR4, and/or OGR1 are expressed in a large number of human cancers, including stomach cancer [52], prostate cancer [150], and CRC [151]. In patients suffering from CRC, GPR4 mRNA and protein are increased as compared to non-tumor tissue, and high expression correlates with late-stage tumors and poor overall survival [151]. In a murine model with subcutaneous HCT116 xenografts, tumor progression was reduced when xenografts were depleted of GPR4 with shRNA [151]. Experiments with GPR4-deficient mice showed reduced tumor angiogenesis and tumor growth in an orthotopic tumor model using the murine colorectal carcinoma cell line CT26 [148]. Histological analysis of tumors indicated altered vessel morphology, length, and density. The reduced angiogenesis is linked to a reduced response of ECs to VEGF, a key mediator of the vascular response to hypoxia [148]. Similarly, intestinal inflammation, development of CRC, and tumor angiogenesis were reduced in GPR4-deficient mice compared to WT control [82].

*Ogr1* mRNA expression is increased in murine colonic tumors compared with normal colonic mucosa [23]. The induction of *Ogr1* in tumor tissue is in good agreement with the known interaction between tumor hypoxia and acidosis [85], and several putative DNA binding sites for HIF-1α within the proximal regions of the *Ogr1* promoter variants have been identified [22].

## Small molecule modulators of pH-sensing receptors may become a therapeutic opportunity for IBD and CRC

The pharmaceutical industry has started to develop modulators of pH-sensing receptors, and information on a few of these molecules is now in the public domain. A group of imidazopyridine derivatives were developed by Novartis, CH as orally active inhibitors of GPR4 and were evaluated for the treatment of inflammatory diseases and pain [86]. Derivative 39c showed promising pharmacokinetic properties (low clearance, good bioavailability, and oral exposure) and was therefore chosen for further profiling. This molecule was tested in an angiogenesis model in rodents and inhibited VEGF-induced angiogenesis in a dose-dependent manner [86]. Importantly, GPR4 antagonist 39c also decreased collagen deposition in a murine model of gut fibrosis [143]. These data further corroborate results from *Gpr4*-deficient mice in models of intestinal fibrosis, indicating that collagen deposition is reduced in the absence of GPR4 signaling. The expression of *Vegfα* mRNA was also decreased upon *Gpr4* antagonist treatment and the presence of shorter vessels and decreased angiogenic factors was indicative of less vascularization. The pathophysiological relevance of GPR4 inhibition was determined in cultured primary human and murine intestinal fibroblasts. An acidic pH shift increased mRNA and protein levels of pro-fibrotic factors as well as stress fiber formation, which were reversed in the presence of the GPR4 antagonist 39c [143]. GPR4 signals via G_12/13_ through GTP-RhoA. RhoA activity increased following acidification in human fibroblasts and decreased upon treatment with the GPR4 antagonist. A close analog of derivative 39c, GPR4 inhibitor compound 13 (also known as NE-52-QQ57), reduced clinical severity and macroscopic disease indicators of intestinal inflammation in a murine acute colitis model [118]. Compound 13 reduced EC activation, leukocyte recruitment into inflamed intestinal tissue, and pro-inflammatory gene expression in the distal colon. Inhibition of GPR4 activity by pharmacological intervention may represent a promising novel approach to reduce inflammation by attenuating vascular EC activation and leukocyte infiltration into inflamed tissues [118].

An OGR1 small-molecule inhibitor (OGR1-I) was developed by Takeda Pharmaceuticals, San Diego, CA, USA. OGR1-I was tested in a murine model of acute colitis and reduced clinical severity [24]. Ameliorated inflammation upon OGR1-I was also demonstrated by endoscopy and histology. Moreover, OGR1-I reduced infiltration of T cells in acute colitis and macrophage recruitment in chronic colitis. OGR-I was also tested in in vitro experiments using the human colon carcinoma cell line Caco-2. These studies showed that endoplasmic reticulum (ER) stress is induced by acidosis-activated OGR1-mediated signaling [80]. ER stress was mediated by OGR1-dependent activation of c-Jun N-terminal kinase (JNK) signaling, the splicing of X-Box binding protein (XBP)1, and the expression of the unfolded protein response marker binding immunoglobulin protein (BiP). Conversely, JNK signaling, XBP1 splicing, and BiP expression were prevented in the presence of the inhibitor OGR1-I. This inhibitor also restored late-stage autophagy blocked by acidic activation of OGR1 in a human intestinal epithelial cell model overexpressing OGR1 [80].

## Summary

Research on proton-sensing GPCRs over recent years has demonstrated that pH variations in a narrow physiological range can actively and specifically influence cell signaling and physiological processes. Progression of IBD is accompanied by low pH and acidosis in the gut; consequently, the three pH-sensing GPCRs, TDAG8, GPR4, and OGR1 play an important role in IBD-associated inflammation, fibrosis, and tumorigenesis.

Anti-inflammatory TDAG8 emerged as a receptor of particular interest because of its genetic link to IBD and other chronic immune pathologies. TDAG8 has a prominent role as a negative regulator of inflammation, due to its cAMP-elevating action in immune cells in response to low extracellular pH.

Pro-inflammatory GRP4 is predominantly expressed on ECs and regulates the expression of endothelial adhesion molecules and angiogenic factors, thereby affecting vascularization and mucosal leukocyte infiltration, key factors in the progress of inflammation. In IBD patients, GPR4 expression is elevated compared to healthy controls, likely reflecting enhanced local angiogenesis and EndoMT in diseased tissue, leading to fibrosis. In patients suffering from CRC, high expression levels of GPR4 correlate with late-stage cancer and poor overall survival.

Pro-inflammatory OGR1 exhibits a pronounced expression in monocytes/macrophages, T cells, granulocytes, ECs, and mesenchymal cells and perpetuates intestinal inflammation through the expression of pro-inflammatory mediators. Moreover, OGR1 appears to play a crucial effector role in inflammation driven by hypoxia and acidosis. As might be expected, the expression of OGR1 correlates with clinical scores given to IBD patients, and samples from fibrotic areas showed increased OGR1 when compared to the non-fibrotic resection margin. An OGR1 small-molecule inhibitor reduced clinical severity in a murine model of acute colitis. Considering the aforementioned, small molecule modulators of pH-sensing receptors constitute a new therapeutic opportunity for IBD and CRC therapy.

Currently, it is unclear how pH-sensing by GPCRs connects with other pH-sensing mechanisms such as certain ion channels and pH-sensitive intracellular enzymes [43]. Work is ongoing to shed more light on this interplay. With respect to therapeutic opportunities, it is noteworthy that GPCRs are expressed on the cell surface and evolved to respond to extracellular cues in very specific ways. For these reasons, they constitute a particularly successful class of molecular targets for therapeutic intervention.

## Data Availability

No datasets were generated or analysed during the current study.

## Abbreviations

BIP: Binding immunoglobulin protein
cAMP: Cyclic adenosine monophosphate
CD: Crohn’s disease
cDNA: Complementary DNA
CRC: Colorectal cancer
DSS: Dextran sodium sulfate
EAE: Experimental autoimmune encephalitis
ECs: Endothelial cells
ECM: Extracellular matrix
EMT: Epithelial-mesenchymal transition
EndoMT: Endothelial-to-mesenchymal transition
FSC: Forward scatter
Gapdh: Glyceraldehyde-3-phosphate dehydrogenase
GPCRs: G protein-coupled receptors
GPR4: G protein-coupled receptor 4
HBSS: Hank’s balanced salt solution
HE: Hematoxylin and eosin
HIF: Hypoxia-inducible factor
IBD: Inflammatory bowel disease
IL: Interleukin
JNK: C-Jun N-terminal kinase
MEICS: Murine endoscopic index of colitis severity;
NFAT: Nuclear factor of activated T cell promoter
OGR1: Ovarian cancer GPR 1 (GPR68)
PBS: Phosphate-buffered solution
qPCR: Real-time quantitative polymerase chain reaction
SSC: Side scatter
TDAG8: T-cell death-associated gene 8 (GPR65)
TME: Tumor-associated extracellular micro-environment
TNF: Tumor necrosis factor
TGF: Transforming growth factor
UC: Ulcerative colitis
VCAM: Vascular cell adhesion molecule-1
VEGF: Vascular endothelial growth factor
WT: Wildtype
XBP: X-box binding protein
